# Supplementary material for: A transcription network underlies the dual genomic coordination of mitochondrial biogenesis
Source: eLife. 2024 Dec 27;13:RP96536. doi: 10.7554/eLife.96536 (PMC11677238; doi:10.7554/eLife.96536)

With Marker

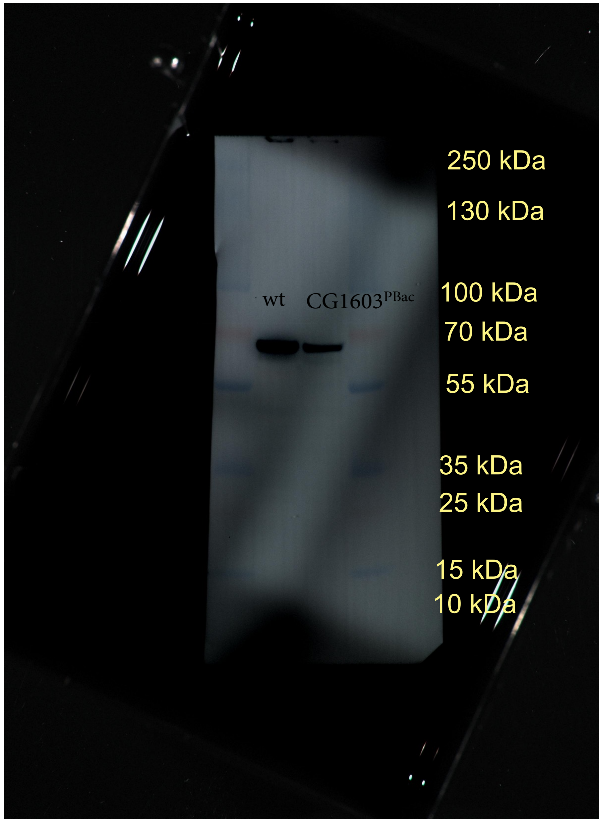

Original

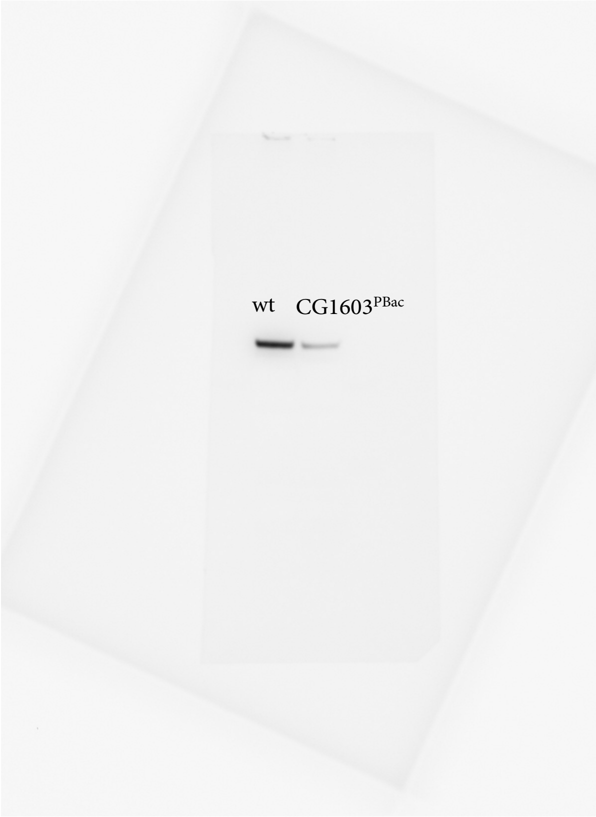

Crop

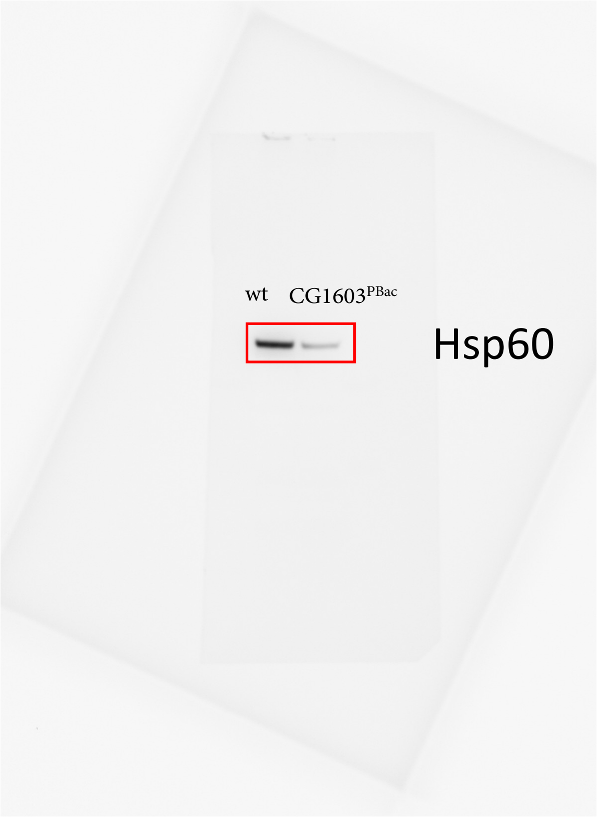

With Marker

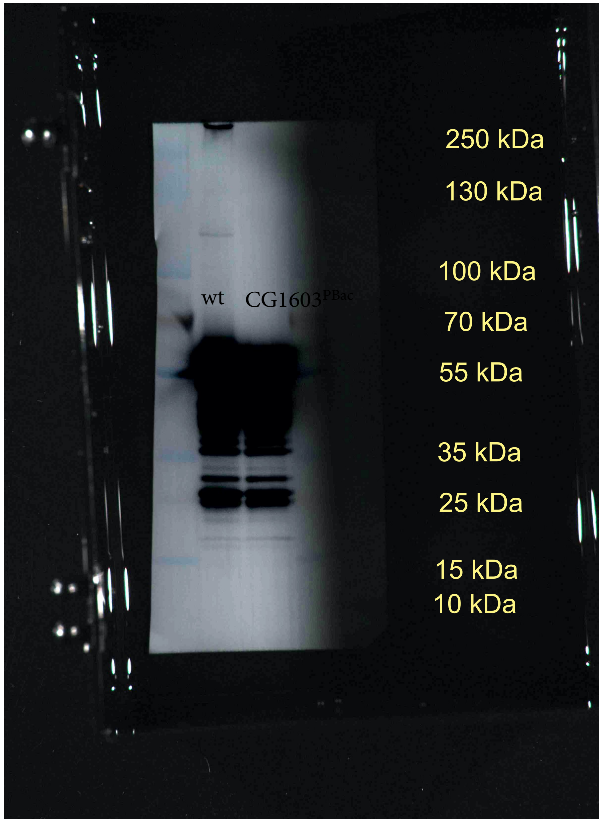

Original

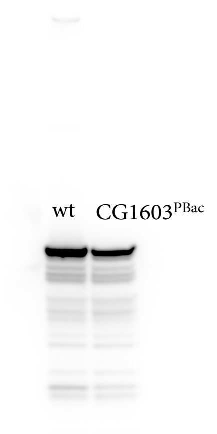

Crop

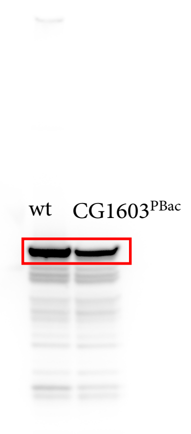

ATP5A

With Marker

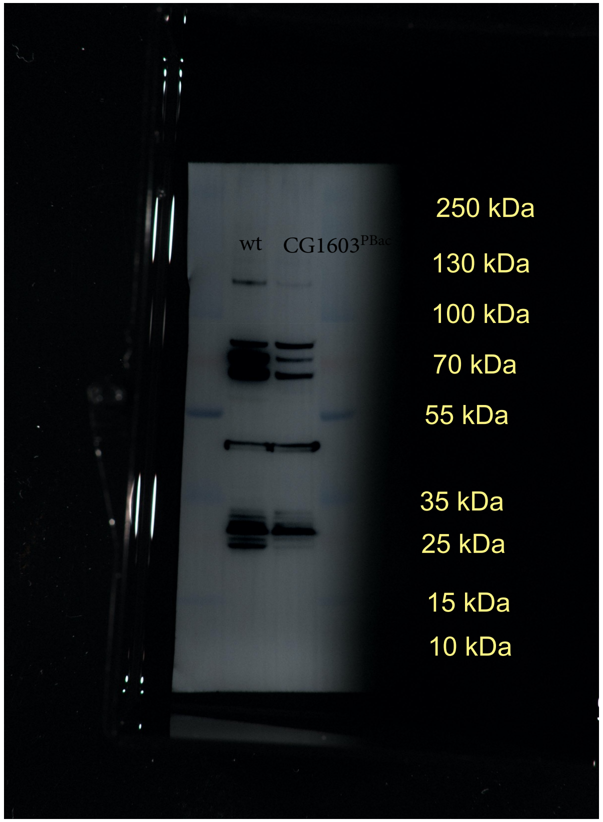

Original

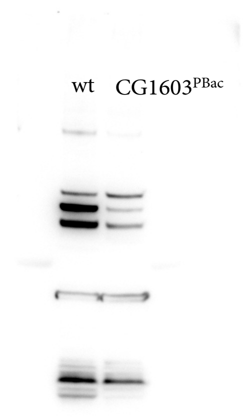

Crop

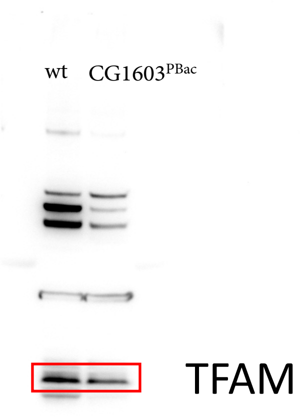

With Marker

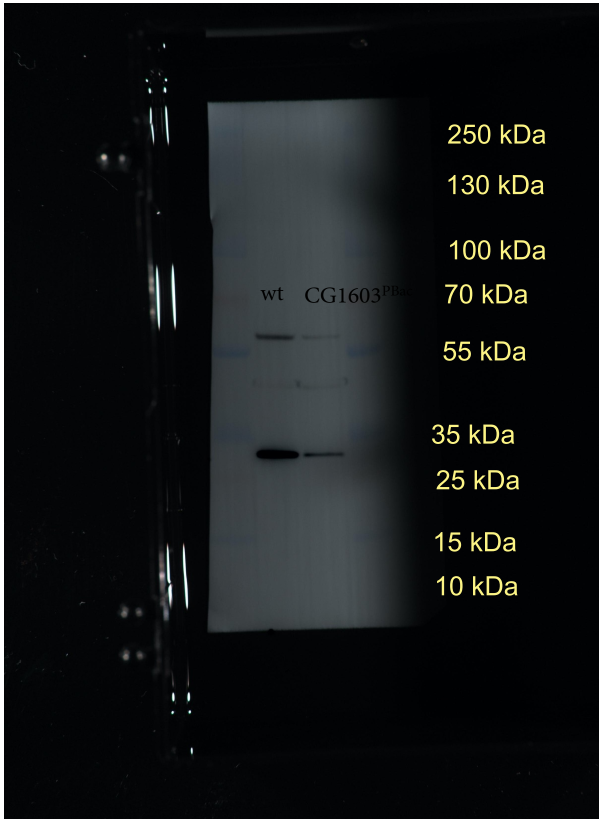

Original

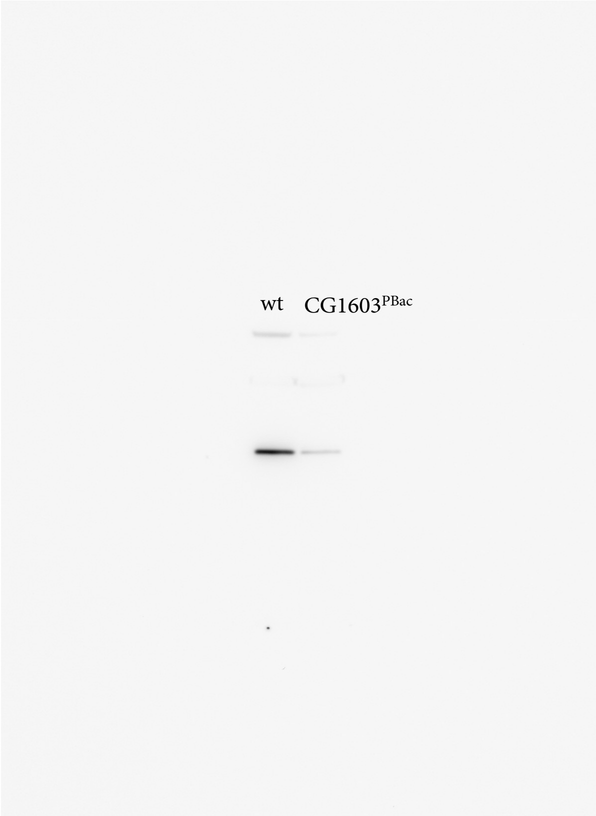

Crop

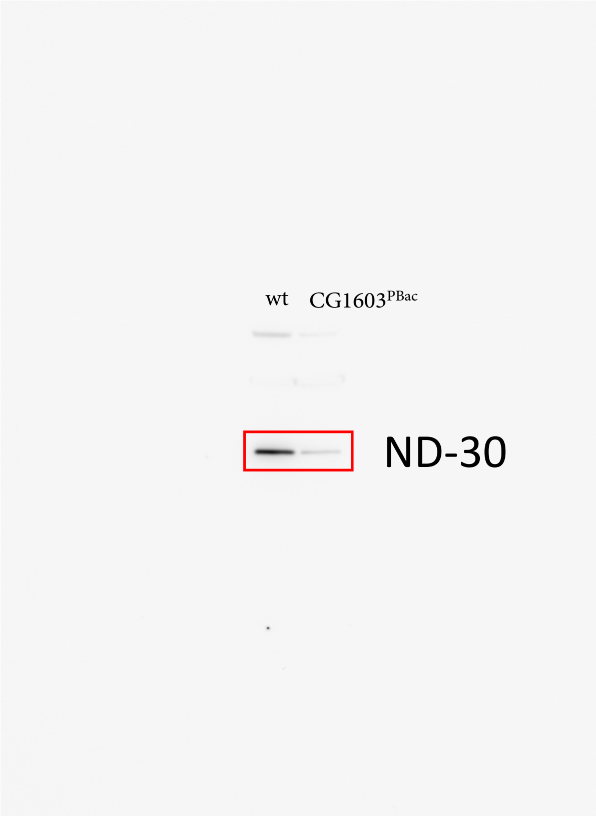

With Marker

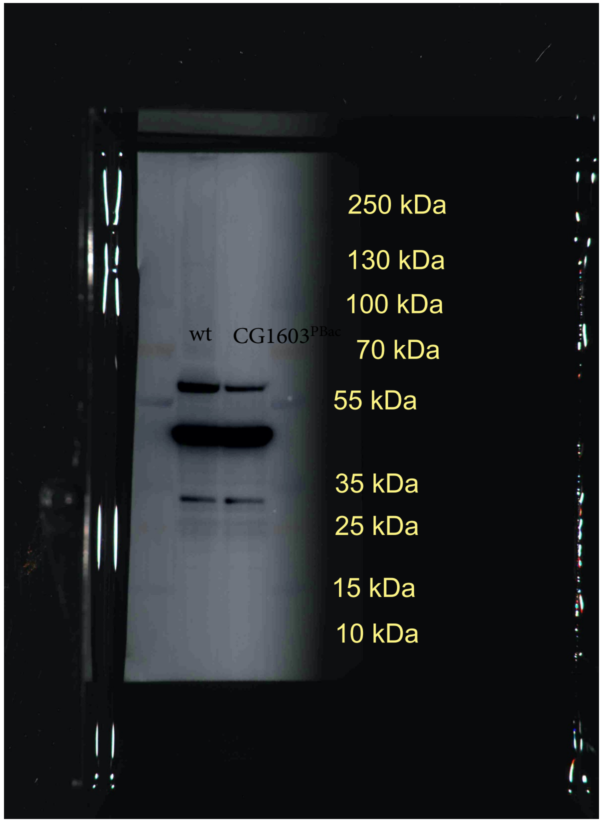

Original

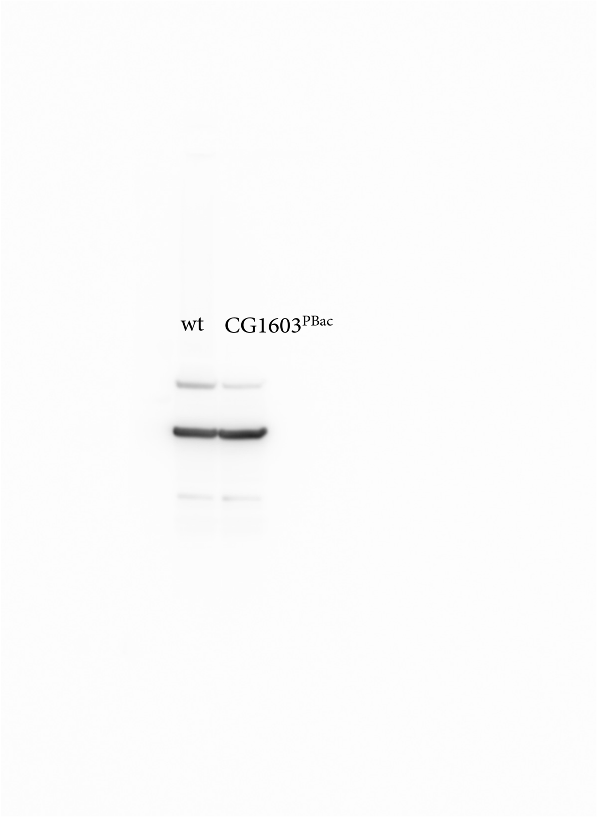

Crop

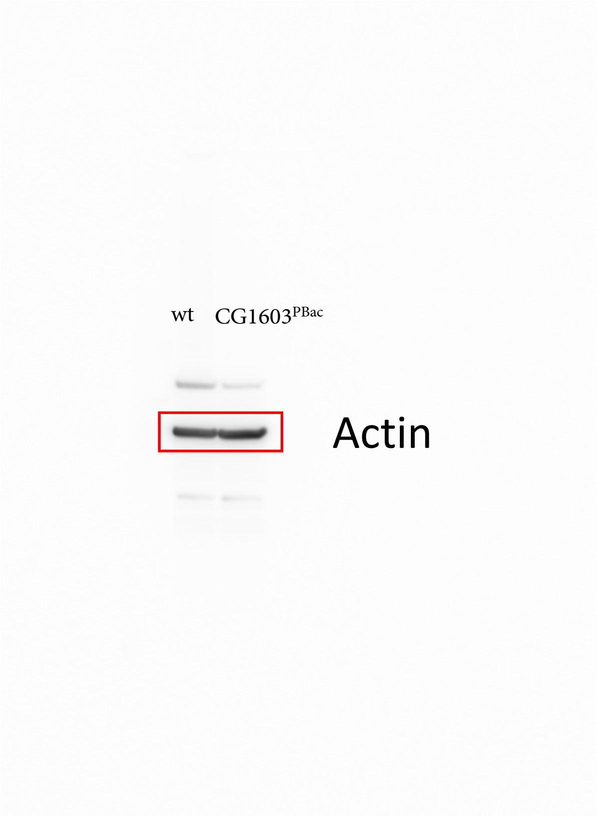

Supplement: Figure 4—source data 2. [file elife-96536-fig4-data2.zip › Figure 4-Source Data 2/Source_data_Figure_4E.pdf]
