# Supplementary material for: A transcription network underlies the dual genomic coordination of mitochondrial biogenesis
Source: eLife. 2024 Dec 27;13:RP96536. doi: 10.7554/eLife.96536 (PMC11677238; doi:10.7554/eLife.96536)
Supplement: Figure 4—figure supplement 1—source data 1. [file elife-96536-fig4-figsupp1-data1.zip › Figure 4-figure supplement 1-source data 1/Source Data_Figure 4_figure supplement 1.pdf]

Complex I

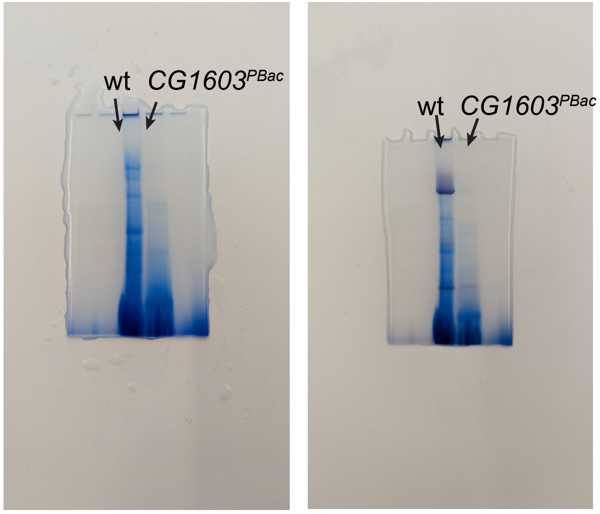

BN-PAGE

BN-PAGE + in-gel activity

Complex II

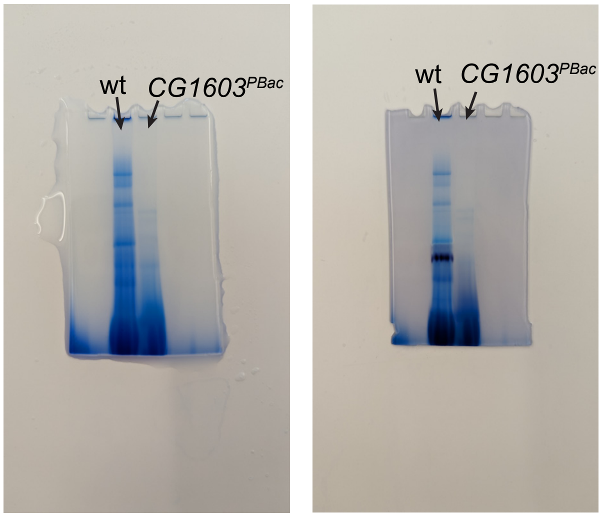

BN-PAGE

BN-PAGE + in-gel activity

Complex IV

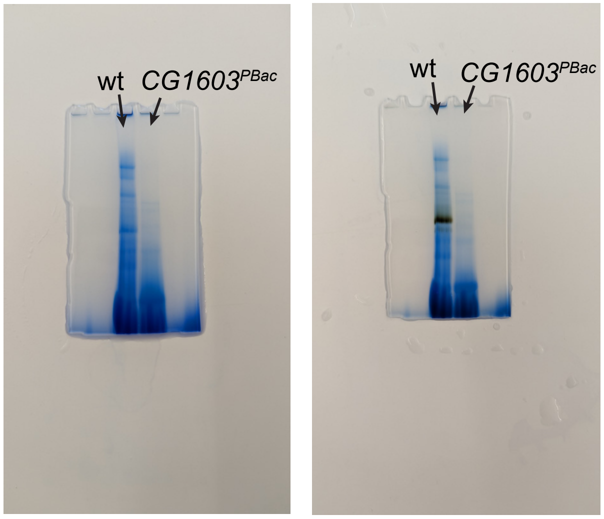

BN-PAGE

BN-PAGE + in-gel activity
