# Supplementary figures and images for: A transcription network underlies the dual genomic coordination of mitochondrial biogenesis
Source: eLife. 2024 Dec 27;13:RP96536. doi: 10.7554/eLife.96536 (PMC11677238; doi:10.7554/eLife.96536)

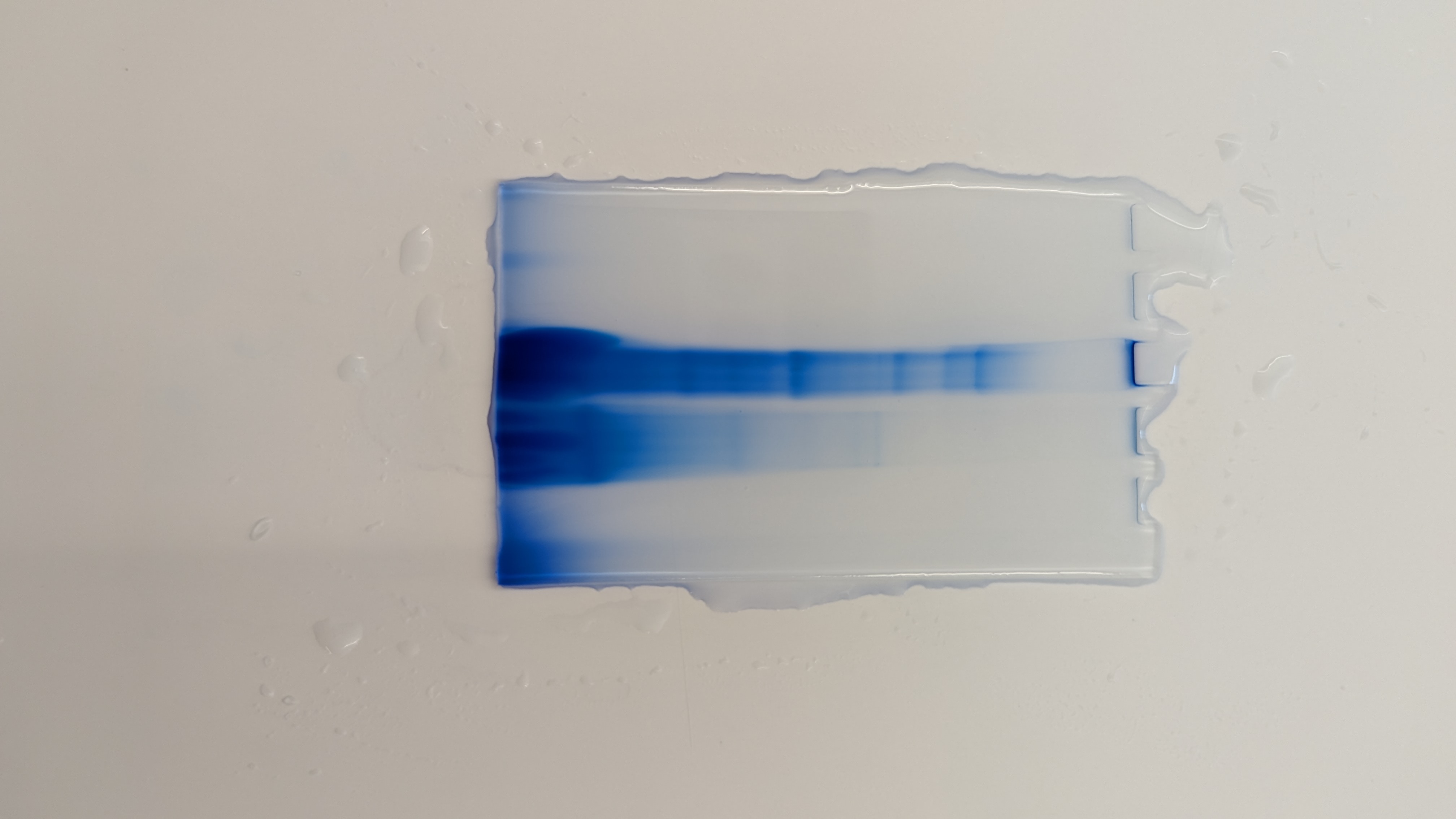

Supplement: Figure 4—figure supplement 1—source data 2. [file elife-96536-fig4-figsupp1-data2.zip › Figure 4-figure supplement 1-source data 2/BN_CI.jpg]

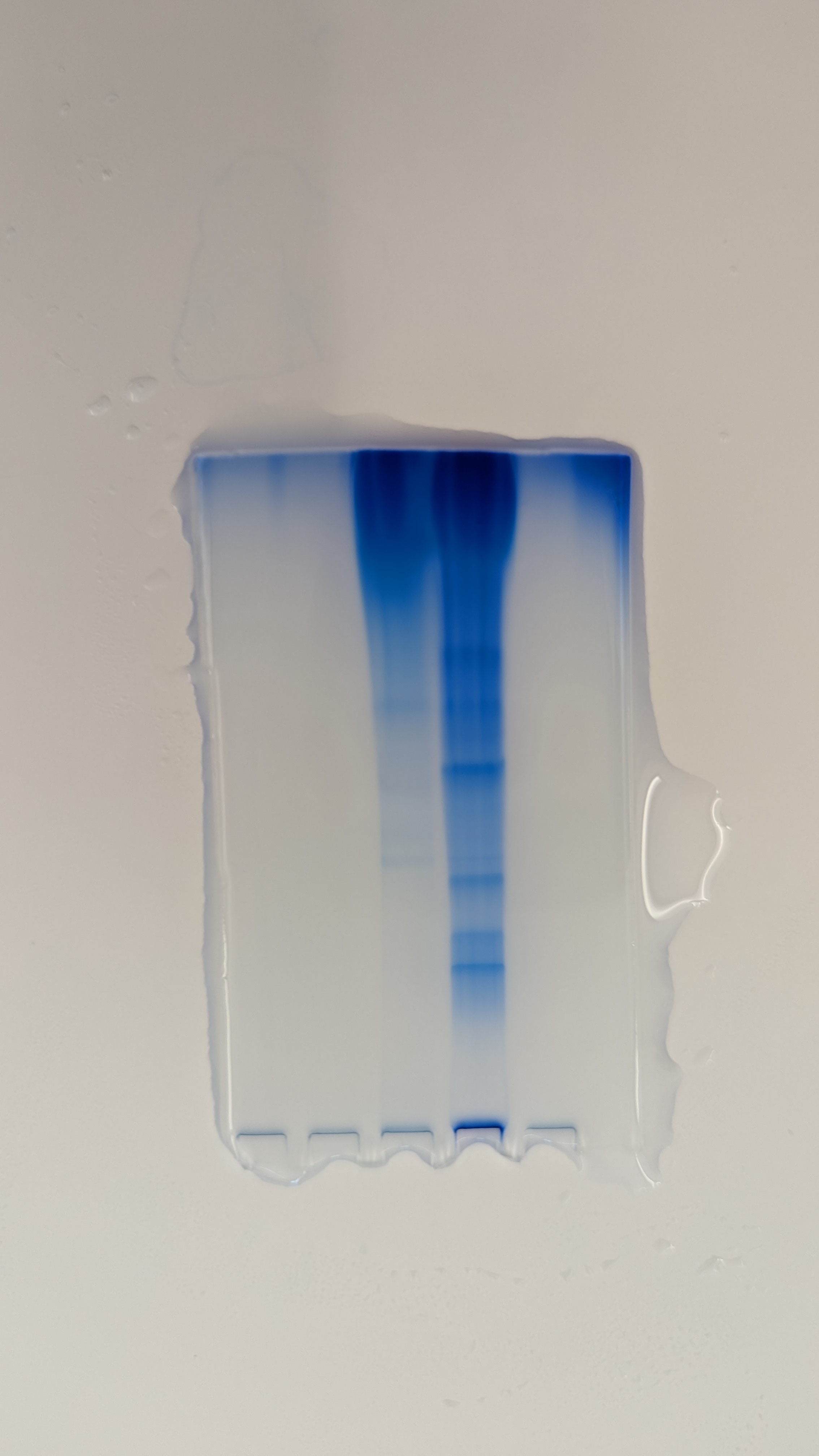

Supplement: Figure 4—figure supplement 1—source data 2. [file elife-96536-fig4-figsupp1-data2.zip › Figure 4-figure supplement 1-source data 2/BN_CII.jpg]

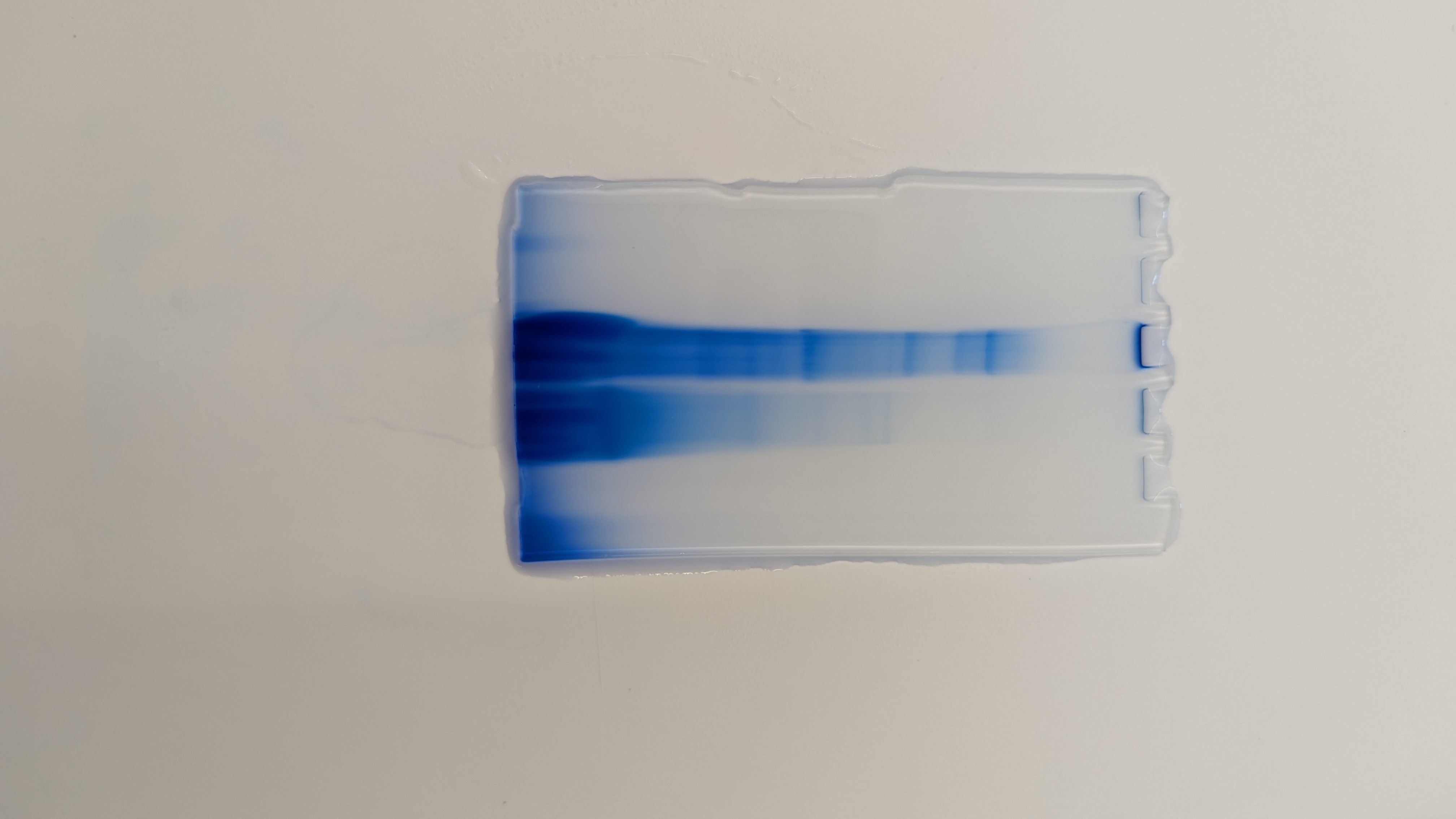

Supplement: Figure 4—figure supplement 1—source data 2. [file elife-96536-fig4-figsupp1-data2.zip › Figure 4-figure supplement 1-source data 2/BN_CIV.jpg]

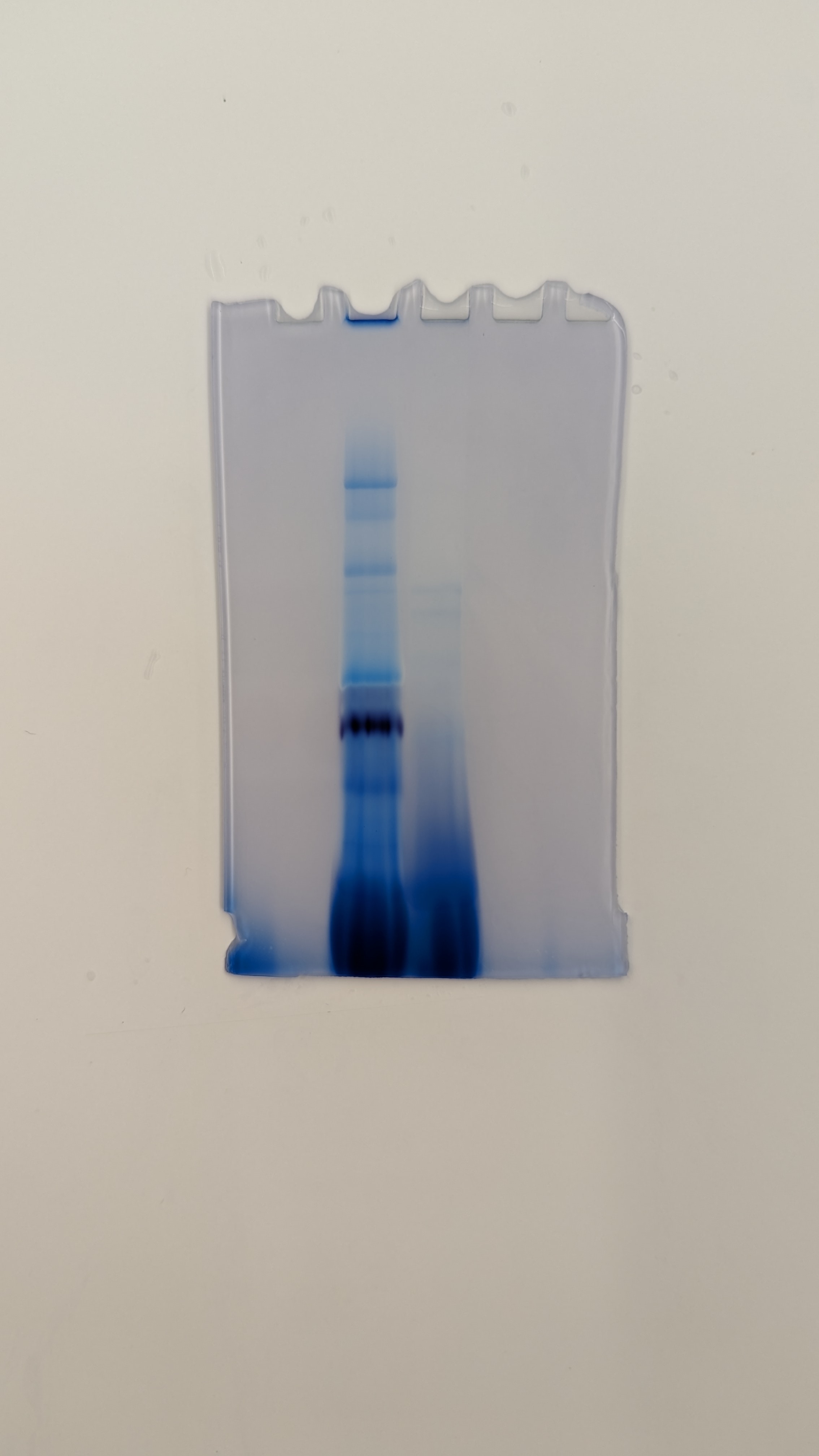

Supplement: Figure 4—figure supplement 1—source data 2. [file elife-96536-fig4-figsupp1-data2.zip › Figure 4-figure supplement 1-source data 2/BN_CII_activity.jpg]

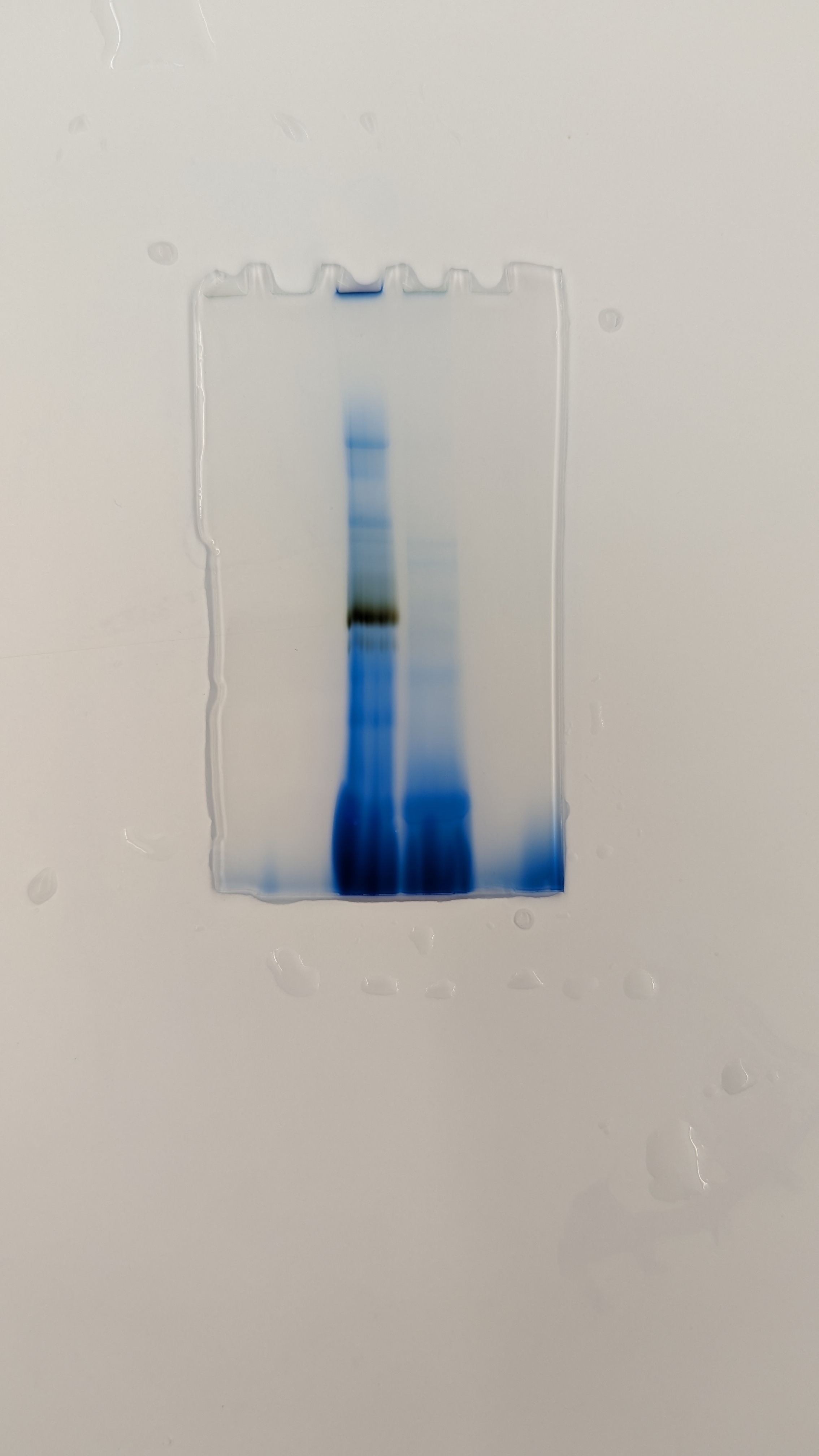

Supplement: Figure 4—figure supplement 1—source data 2. [file elife-96536-fig4-figsupp1-data2.zip › Figure 4-figure supplement 1-source data 2/BN_CIV_activity.jpg]

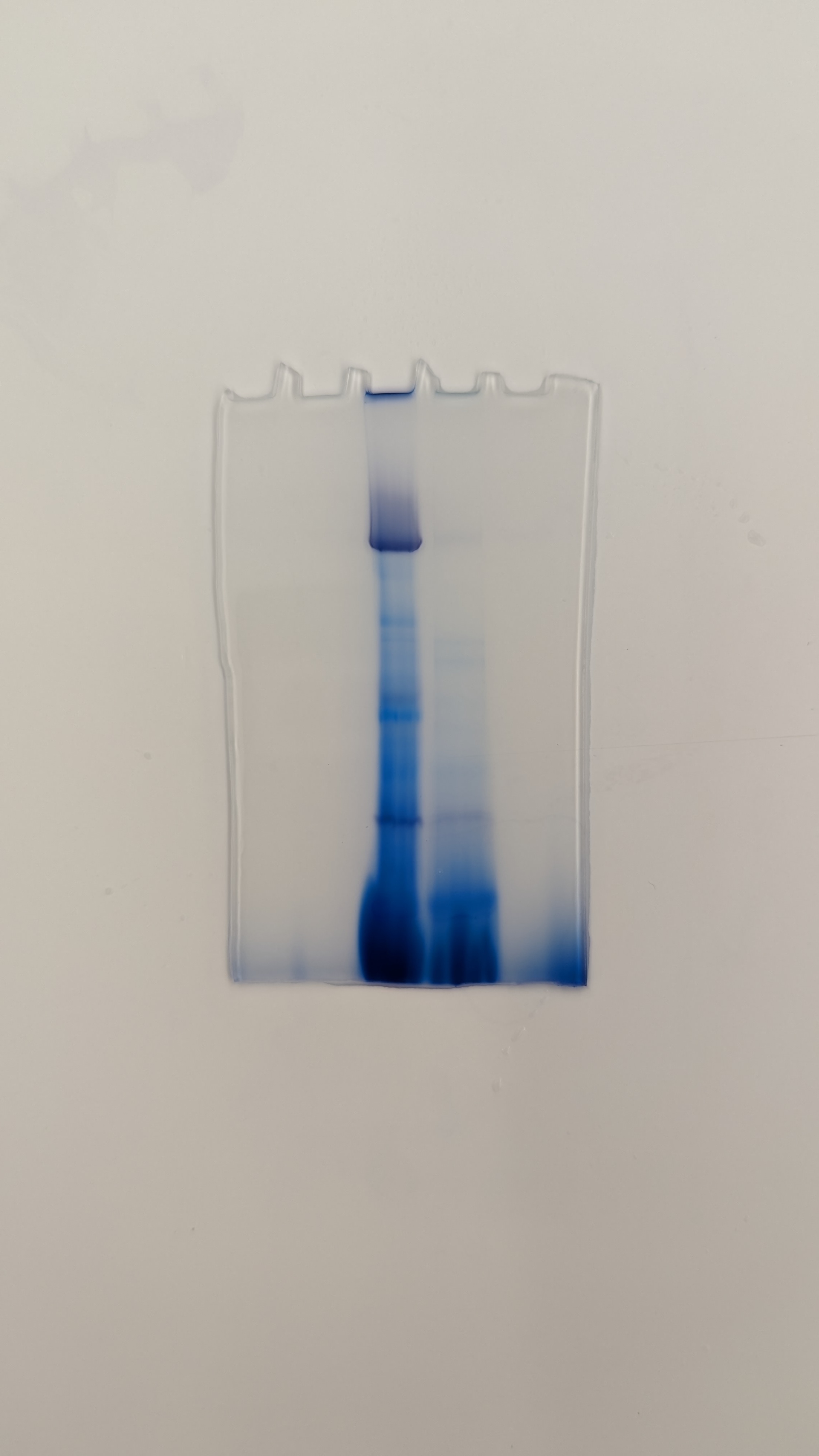

Supplement: Figure 4—figure supplement 1—source data 2. [file elife-96536-fig4-figsupp1-data2.zip › Figure 4-figure supplement 1-source data 2/BN_CI_activity.jpg]
